# Bayesian sequential monitoring strategies for trials of digestive cancer therapeutics

**DOI:** 10.1186/s12874-024-02278-3

**Published:** 2024-07-19

**Authors:** Guillaume Mulier, Ruitao Lin, Thomas Aparicio, Lucie Biard

**Affiliations:** 1 ECSTRRA team UMR 1153, INSERM, Saint-Louis hospital, 1 avenue Claude Vellefaux, Paris, 75010 France; 2 Service de Biostatistique et Information Médicale, AP-HP Saint-Louis hospital, 1 avenue Claude Vellefaux, Paris, 75010 France; 3 Department of Biostatistics, MD Anderson Cancer Center, 7007 Bertner Avenue, Houston, 77030 Texas USA; 4 Service d’hépato-gastro-entérologie, Hôpital Saint-Louis, 1 avenue Claude Vellefaux, Paris, 75010 France; 5 Université Paris Cité, 12 rue de l’École-de-Médecine, Paris, 75006 France

**Keywords:** Phase II, Adaptive design, Bayesian, Oncology

## Abstract

**Background:**

New therapeutics in oncology have presented challenges to existing paradigms and trial designs in all phases of drug development. As a motivating example, we considered an ongoing phase II trial planned to evaluate the combination of a MET inhibitor and an anti-PD-L1 immunotherapy to treat advanced oesogastric carcinoma. The objective of the paper was to exemplify the planning of an adaptive phase II trial with novel anti-cancer agents, including prolonged observation windows and joint sequential evaluation of efficacy and toxicity.

**Methods:**

We considered various candidate designs and computed decision rules assuming correlations between efficacy and toxicity. Simulations were conducted to evaluate the operating characteristics of all designs.

**Results:**

Design approaches allowing continuous accrual, such as the time-to-event Bayesian Optimal Phase II design (TOP), showed good operating characteristics while ensuring a reduced trial duration. All designs were sensitive to the specification of the correlation between efficacy and toxicity during planning, but TOP can take that correlation into account more easily.

**Conclusions:**

While specifying design working hypotheses requires caution, Bayesian approaches such as the TOP design had desirable operating characteristics and allowed incorporating concomittant information, such as toxicity data from concomitant observations in another relevant patient population (e.g., defined by mutational status).

**Supplementary Information:**

The online version contains supplementary material available at 10.1186/s12874-024-02278-3.

## Background

The development of drugs in oncology has long driven statistical innovations for trial designs [1, 2]. The severity of the disease has led to considering drugs with non-negligible toxicity [3, 4]. This particular benefit/risk ratio results in trials enrolling patients, from the early phases of development, rather than healthy volunteers [5]. Methodological challenges have arisen over the past decades with new types of treatments such as immunotherapy [6]. Their mode of action differs from that of conventional cytotoxic chemotherapy, resulting in different kinetics in both toxicity and efficacy, often with prolonged timeframes. Such drugs have a longer duration of treatment and prolonged effect after cycles, as opposed to intermittent action after cycles for cytotoxic chemotherapy.

Phase II trials, notably in oncology, aim at identifying promising therapies while ruling out the unpromising as soon as possible, prior to large-scale phase III studies [7]. Designs for phase II trials encompass a wide range of approaches and sample sizes, including randomization with a control arm [8, 9]. We focused our work on single-arm phase II trials only, which remain commonplace in oncology. Furthermore, designs with sequential monitoring of efficacy or toxicity are the choice in phase II, allowing early decisions: stopping for futility or graduating early promising treatments [10]. However, some features of immunotherapy trials have challenged the existing sequential monitoring approaches. Specifically, immunotherapy usually implies long-term endpoints which may reduce the feasibility of frequent interim analyses [6, 11–13].

Also, nowadays, it is more and more common to perform a cohort expansion after phase I trial, giving potentially more information about efficacy and toxicity for phase II trials [14]. Moreover, sample sizes are often limited, and designs may include the joint evaluation of efficacy and toxicity to some extent. Overall, all these particularities may result in the need for complex designs, and multiple analyses may be combined with caution to avoid an inflated risk of false positive [15–17]. Lastly, endpoints are often assessed in a shorter time compared to phase III trials. For efficacy, RECIST criteria are allowing a standardized way of assessing the ORR as a categorical variable in cancer treatment, primarily for early phase II clinical trials [18]. These criteria were developed for chemotherapeutic agents, and thus for immunotherapies, iRECIST criteria were developed [19]. Similarly, the evaluation of the toxicity endpoint is standardized via classifications like NCI-CTCAE or specific scales for targeted toxicities.

In this paper, we took as an example the statistical planning of the single-arm phase II METIMGAST trial. Then we consider several design options given the specific clinical settings and compare their operating characteristics in a simulation study. The aim is to investigate if the adding of a toxicity monitoring independently from a monitoring design for efficacy led to desirable properties, and if using a design like TOP using pending patient information could have advantages over designs using brute counts and to exemplify the specific challenges during the planning of such a trial. Lastly, we provide points for discussion.

## Methods

### Motivating example

The single-arm phase II METIMGAST trial (NCT05135845) assessed the combination of capmatinib and spartalizumab in advanced oesogastric adenocarcinoma in adults. Spartalizumab is an anti-PDL1 monoclonal antibody, and capmatinib is a tyrosine kinase inhibitor targeting the c-MET receptor. The combination has in vitro evidence of a synergistic action of the two molecules [20, 21] and has recently been evaluated in lung, breast, and liver cancer. An adaptive design was planned (see later) with sequential monitoring rules for efficacy and toxicity with 90 patients. The two endpoints used for monitoring were the objective response rate (ORR) for efficacy in MET-negative patients (81 expected) and the occurrence rate of an unacceptable toxicity in the whole set of patients. The ORR is defined as the proportion of patients with partial or complete remission according to RECIST v1.1 criteria within 6 months after inclusion (that is 8 cycles of treatment); and the occurrence of an unacceptable toxicity event was captured within 42 days after inclusion (corresponding to 2 cycles of treatment), defined using NCI-CTCAE v5 criteria.

### The planned design

The primary observation window was planned at 6 months from inclusion, and the anticipated accrual rate was 5 patients per month. The trial was therefore initially planned using an adaptive Bayesian phase 2 design with sequential analyses, allowing continuous recruitment: the Time-to-event Optimal Phase 2 design [22], for efficacy analyses in MET-negative patients, with interim at 30 and final at 81 patients. The TOP design is derived from the BOP2 design [23], which is a clinical trial design under a Bayesian framework. It models unique or multiple endpoints (efficacy alone, efficacy + toxicity, for example) through a multinomial distribution and provides decision rules to stop the trial. Posterior probabilities of the probability of an endpoint being inferior to a prespecified critical value $\phi$ (elicited with clinicians) are computed at each analysis: $Pr(p\le \phi |D_n)$ with *p* the probability of the endpoint of interest and $D_n$ the data available at interim analysis. Then, these posterior probabilities are compared to a threshold for stopping decision rules, $Pr(p\le \phi |D_n)>C_n$, where $C_n$ takes the form of a power function: $C_n=1-\lambda \left(\frac{n}{N}\right)^\gamma$ with *n* the number of recruited patients, *N* the maximum number of patients, and $\lambda$ and $\gamma$ 2 hyperparameters optimized before the trial. BOP2 design uses counts, and TOP design extends to long-term outcomes by taking into account the information of pending patients via a weighting in the likelihood, allowing to shorten the duration of trials.

The TOP design was used for efficacy assessment only in METIMGAST trial. Working hypotheses on efficacy were the following: $H_0: p_{\text {0,eff}}\le 0.15$ and $H_1: p_{\text {1,eff}}=0.30$. The design was parameterized to ensure 90% power under these hypotheses, given a 5% type I error rate with 90 patients and an accrual of 5 patients per month. The design’s parameters were $\lambda =0.92/\gamma =0.97$, which provided 94.76% power under $H_1$.

Motivated by safety concerns, toxicity monitoring was added with more frequent looks (at 5, 10, 15, 20, 30, 40, 50, 60, 70, 80, and 90 patients over both groups). This toxicity monitoring was planned using a posterior distribution approach [24, 25] with the following decision rule: $Pr(p_{\text {tox}}>0.25|D_n)>0.95$, where $p_{\text {tox}}$ is the probability of unacceptable toxicity. By simulation, it was assessed that the addition of the proposed toxicity monitoring would keep power above 90% when toxicity risk is low ($p_{\text {tox}}\le 0.20$) and that in the case of unacceptable toxicity, the trial will be stopped in 49% of cases when $p_{\text {tox}}=0.30$ and 95% when $p_{\text {tox}}=0.40$. Retrospectively, to ensure comparability with other designs, we changed the parameters of TOP design ($\lambda = 0.865 / \gamma = 0.91$) to ensure a 10% type I error rate for the TOP design. By simulation, it was assessed that the resulting design combining both stopping rules had a type I error rate of 4.43% under the aforementioned efficacy and toxicity rates with a slight positive correlation between them, and a power of 94.25% (2.6% and 92.5% respectively for the initial design).

Of note, a simple estimation of ORR was planned in MET-positive patients, without interim analysis, independently of analyses in MET-negative patients.

### Studied designs

The aim of the present work was to compare several approaches to trial design in the setting of the METIMGAST study, that is a single-arm phase II trial with an interim analysis with a futility stopping rule and allowing toxicity monitoring including stopping rules as well. Overall, we compared the main proposal described above (denoted “TOP$_\text {eff}$+PP$_\text {tox}$” hereafter) with four other approaches adapted to our clinical setting in terms of trial design. We evaluated designs omitting the time-to-event information on the outcomes using two designs with strictly binary endpoints, and two approaches relying on variations of the TOP design. For all approaches, sample sizes (interim and final) for efficacy analyses were kept identical to the original proposal: 30 and 81 patients.

The initial METIMGAST trial aimed at evaluating the therapeutic effect of the capmatinib-spartalizumab combination, relying on the assumption of a synergistic activity of the drugs, rather than relying on the cMET inhibition itself with capmatinib. To that aim, the target population of the trial, for efficacy assessment, was patients without cMET-amplification, who correspond to the majority of patients (90%). Nevertheless, the remaining 10% of patients, with a cMET amplification, were planned to be eligible as well for the trial, as exploratory analysis. Moreover, since toxicity of the combination was not anticipated to be dependent on cMET-amplification, observed data on the small cohort of c-MET positive patients were considered as informative on the toxicity profile of the treatment overall. The main efficacy analysis was planned on the expected 81 cMET negative patients and toxicity analyses included all 90 patients, cMET negative and positive. We defined 4 trial designs (approaches 1 to 3, and 5) consistent with these clinical settings, and one other (approach 4) in a simpler setting assuming a homogeneous trial population including only c-MET negative patients.

Overall, five approaches were evaluated:

1. TOP design to assess the efficacy, associated with parallel toxicity monitoring based on a posterior probability rule at 5/10/15/20/30/40/50/60/70/80/90 patients “TOP$_\text {eff}$+PP$_\text {tox}$”).
2. Simon’s 2 stage design [26] to assess the efficacy, associated with parallel toxicity monitoring based on a posterior probability rule at 5/10/15/20/30/40/50/60/70/80/90 patients (referred to as “Simon + PP$\text {tox}$” in the following).
3. BOP2 design for efficacy [23] with interim analyses at 30 and 81 patients and the posterior probability’s approach for toxicity assessment, at the same numbers of patients as “TOP$_\text {eff}$+PP$_\text {tox}$” (denoted “BOP$_\text {eff}$+PP$_\text {tox}$” hereafter). Because of the binary definition of BOP2, the accrual is suspended until all observation windows are completed.
4. TOP design with co-primary monitoring endpoints, efficacy and toxicity, with analyses at 30 and 81 patients for efficacy and at (5/10/15/20/30/40/50/60/70/81) patients for toxicity, using only MET-negative patients (denoted “TOP$_\text {eff/tox}^t$” below);
5. TOP design with co-primary monitoring endpoints, efficacy and toxicity, with analyses at 30 and 81 patients for efficacy and at (5/10/15/20/30/40/50/60/70/81) patients for toxicity, in MET-negative patients, but incorporating accumulated data from MET-positive patients, as it becomes available for the assessment of toxicity. It results an informative prior for toxicity rate’s posterior distribution (details in Additionnal file 1 section 3) derived from information on toxicity in MET-positive patients by assuming the homogeneity of toxicity regarding MET status (referred to as “iTOP$_\text {eff/tox}$” hereafter).

### Design calibration

All designs were calibrated based on the probability of conclusion to a promising treatment under the following working null and alternative hypotheses with a positive correlation between efficacy and toxicity: $H_0:\{p_{\text {0,eff}}=0.15; p_{\text {0,tox}}=0.30;$ correlation coefficient between efficacy and toxicity: $R=0.21\}$ and $H_1:\{p_{\text {1,eff}}=0.30; p_{\text {1,tox}}=0.20; R=0.26\}$.

Decision boundaries for the “TOP$_\text {eff}$+PP$_\text {tox}$” and “BOP$_\text {eff}$+PP$_\text {tox}$” were tuned following the BOP2/TOP procedure for efficacy [22, 23] and using the posterior distribution on toxicity risk [24, 25] for toxicity, under the above-listed hypotheses on efficacy and toxicity; parameters for the BOP2/TOP efficacy decision boundaries were $\lambda =0.865/\gamma =0.91$ (using notations from the original TOP paper). PP$_\text {tox}$ design was added with a stopping rule determined as described in “The planned design” section, and decision rules are of the form of maximal counts of toxicity to continue the trial.

The designs “TOP$_\text {eff/tox}^t$” and “iTOP$_\text {eff/tox}$” used the same decision boundaries formula. Calculation of $\lambda$ and $\gamma$ was adapted for these approaches (see Additional file 1 section 1 for details) and we obtained: $\lambda =0.69/\gamma =0.98$. They corresponded to a type I error rate of 3.79% and a power of 89.27%.

Lastly, for comparative purposes, we also implemented a Simon’s two-stage design minimizing the average sample size under $p_{\text {0,eff}}=0.15$ with the first analysis at 30 patients and the final at 81 patients. This design calibration is described in Additional file 1 section 1. Decision boundaries in terms of efficacy and toxicity event counts are available in Additional file 1 section 4 for all designs.

Of note, when adding PP$_\text {tox}$ monitoring on top of an efficacy design, it is not straightforward to take into account the correlation between efficacy and toxicity since each endpoint is being handled separately in distinct independent models. In efficacy-toxicity based designs (TOP with co-primary endpoints), the correlation is directly handled via multinomial modeling in design calibration. The pair of calibration parameters $(\gamma , \lambda )$ for the BOP2/TOP approaches may vary depending on the assumed correlation between efficacy and toxicity. In the original trial design, efficacy and toxicity were considered independent, but a probable hypothesis would be that efficacy and toxicity are positively correlated [27, 28].

### Simulation settings

We evaluated the operating characteristics of the five designs under 10 scenarios of true efficacy, $p_\text {eff}$, and toxicity, $p_\text {tox}$, as reported in Table 1. For each scenario, correlation variations were specified, exploring the range of possible correlation between efficacy and toxicity, from $R_{\text {min}}$ (that is when $Pr(\text {Eff}\cap \text {Tox}) = \max (0, Pr(\text {Eff})+Pr(\text {Tox})-1)$) to $R_{\text {max}}$ (that is when $Pr(\text {Eff}\cap \text {Tox}) = \min (Pr(\text {Eff}), Pr(\text {Tox}))$). Details on the different correlations explored are available in Additionnal file 1 section 2.

**Table 1:** Simulation scenarios, with $Pr(\text {Eff})$ as the true probability of efficacy, $Pr(\text {Tox})$ as the true probability of toxicity, $R_\text {min}$ and $R_\text {max}$ as the true minimum and maximum correlations respectively, given the event probabilities

Scenario	Description	$Pr(\text {Eff})$	$Pr(\text {Tox})$	$R_\text {min}$	$R_\text {max}$
1	$H_0$	0.15	0.30	-0.27	0.64
2	$H_1$	0.30	0.20	-0.33	0.76
3	Intermediate	0.20	0.25	-0.29	0.87
4	Intermediate (2)	0.25	0.25	-0.33	1.00
5	Inefficacious	0.15	0.20	-0.21	0.84
6	Inefficacious (2)	0.10	0.15	-0.14	0.79
7	Intermediate efficacy	0.20	0.20	-0.25	1.00
8	Intermediate efficacy (2)	0.20	0.30	-0.33	0.76
9	Toxic	0.30	0.30	-0.43	1.00
10	Toxic (2)	0.40	0.35	-0.60	0.90

For example, scenario 1 with correlation $R_\text {pos,1}$ corresponds to $\{Pr(\text {Eff}\cap \text {Tox})=0.08$, $Pr(\text {Eff}\cap \overline{\text {Tox}})=0.07$, $Pr(\overline{\text {Eff}}\cap \text {Tox})=0.22$, $Pr(\overline{\text {Eff}}\cap \overline{\text {Tox}})=0.63\}$ and scenario 2 to $\{Pr(\text {Eff}\cap \text {Tox})=0.11$, $Pr(\text {Eff}\cap \overline{\text {Tox}})=0.19$, $Pr(\overline{\text {Eff}}\cap \text {Tox})=0.09$, $Pr(\overline{\text {Eff}}\cap \overline{\text {Tox}})=0.61\}$. For the sake of simplicity, results with a positive correlation between efficacy and toxicity ($R_\text {pos,1}=\frac{\text {R}_{\text {max}}}{3}$) are primarily reported in the following section. We also presented the results of the 6 correlations presented in Additional file 1 applied to scenarios 1 and 2 to assess the risk of false positive conclusions under the inefficacy and toxicity case, and the risk of false negative under the case of desirable efficacy and unacceptable toxicity. Additional results on the other scenarios are available in Additional file 1 section 5.

For each scenario, 10000 simulated trials of 90 patients were generated, following the desired sample size for METIMGAST trial, with designs calibrated for a 5% type I error rate. The observation windows were 180 days for efficacy (8 cycles of treatment) and 42 days for toxicity (2 cycles of treatment). The anticipated accrual rate was 5 patients per month, that is a mean interpatient arrival time of 6 days. We estimated the following characteristics for each candidate design: probability of conclusion of efficacy and acceptable toxicity (positive trial), average trial duration, average sample size, probability of early stopping. All analyses were performed on R statistical software version 4.0.2.

## Results

**Fig. 1:**
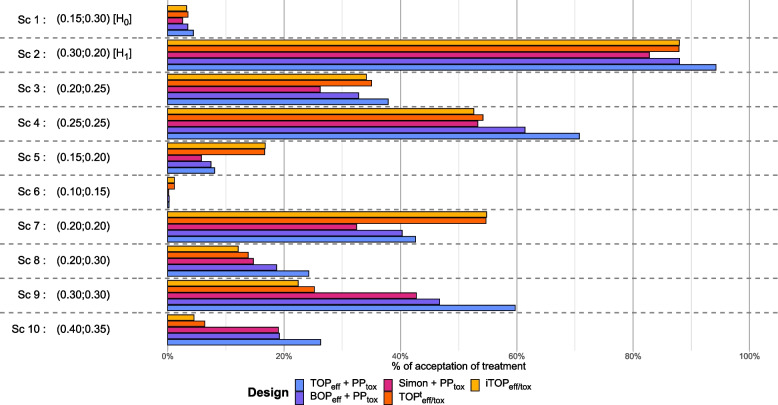
Percentage of conclusions regarding efficacy and acceptable toxicity in the 10 scenarios with a positive correlation between efficacy and toxicity. Numbers in parentheses represent $p_\text {eff}$ and $p_\text {tox}$ for each scenario

Figure 1 represents the percentage of conclusions drawn for a promising treatment (efficacy and acceptable toxicity) across the 10 scenarios with a positive correlation between efficacy and toxicity (ranging from 0.21 to 0.33 depending on the scenario, see Table 1 for details).

Results from scenario 1 showed that the risk of false positives is controlled under $\{p_{\text {0,eff}}=0.15; p_{\text {0,tox}}=0.30\}$, regardless of the design, with Simon + PP$_\text {tox}$ design being the most conservative. Scenario 2 allowed assessing the power under the anticipated $\{p_{\text {1,eff}}=0.30; p_{\text {1,tox}}=0.20\}$: power was the greatest using the TOP$_\text {eff}$ + PP$_\text {tox}$ approach (94.25%); Simon + PP$_\text {tox}$ approach appears the most conservative (82.79%). The remaining 3 approaches have similar power (87.99% for BOP$_\text {eff}$ + PP$_\text {tox}$; 87.94% for iTOP$_\text {eff/tox}$; 87.86% for TOP$_\text {eff/tox}^t$). With intermediate efficacy and toxicity (Sc 3 and 4), TOP$_\text {eff}$ + PP$_\text {tox}$ and BOP$_\text {eff}$ + PP$_\text {tox}$ show higher rate of acceptance. Of note, for treatment with discordant profiles of efficacy and toxicity (efficacious and toxic or not efficacious and not toxic), the more the rate is far from $H_0$, the more the risk of false positive decreases. When the treatment is ineffective, the two TOP$_\text {eff/tox}$ approaches have higher rate of false positive, while when the treatment is toxic these 2 approaches have lower rate of false positive.

Lastly, the approach including the update of the toxicity prior using data from MET-positive patients allowed slightly better control of the risk of false positives compared to the other TOP-only strategy.

The average number of patients per trial, the proportion of early stopping, and the mean duration of a trial with a slight positive correlation between efficacy and toxicity are represented in Additional file 1 section 5.

Overall, the approach Simon + PP$_\text {tox}$ resulted in a higher proportion of early stopping in any scenario. Consistently, the sample size was smaller with this design.

Regarding the remaining four designs, the update of the prior and TOP$_\text {eff/tox}^t$ strategies tend to have a greater proportion of early stopping (more pronounced with the update of the prior), consistent with a reduced risk of false positives and a reduced sample size.

The average duration of a trial was greater with the BOP$_\text {eff}$+PP$_\text {tox}$ and Simon + PP$_\text {tox}$ designs, which was expected given that these designs require waiting for complete observation of all included patients at each interim analysis. For the remaining three designs, the duration was consistent with the mean number of patients per trial.

**Fig. 2:**
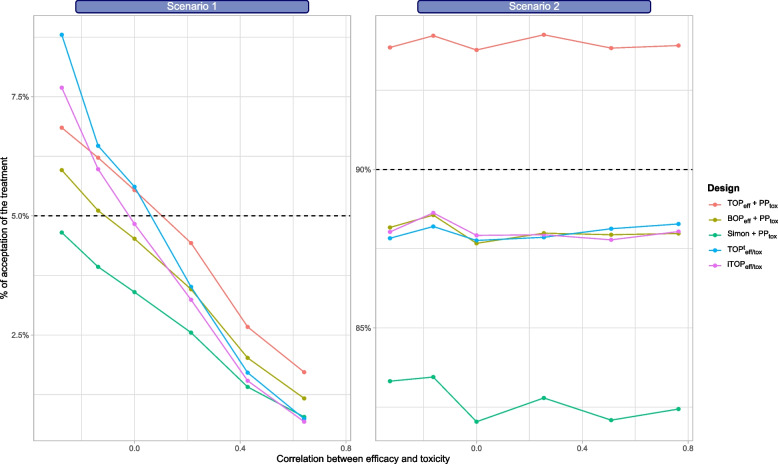
Percentage of conclusions regarding efficacy and acceptable toxicity relative to the correlation between efficacy and toxicity in the setting. Left panel: type I error rate; Right panel: power. (Scale is panel-specific)

We then assessed if the approaches were robust to discrepancies between planning hypotheses and reality in terms of correlation between efficacy and toxicity. Figure 2 presents the type I error rate and power when the correlation between efficacy and toxicity in the trial varies.

Even though the TOP design directly accounts for the correlation between efficacy and toxicity when planning the trial using a multinomial sampling distribution, its type I error rate was impacted by miss-specified correlation. The approaches combining a monitoring for efficacy associated with the posterior probabilities’ approach showed the same pattern. Specifically, for all designs, the type I error rate increased when the correlation was smaller. Power remained about constant for all designs. Additional results on the correlation between efficacy and toxicity in the different scenarios are presented in Additional file 1 section 5.

## Discussion

We report the comparative study of several approaches of trial design for a phase II clinical trial with practical and specific challenges. More specifically, our work was motivated by a phase II clinical trial evaluating a new combination of drugs in the treatment of oesogastric cancer with additional information on toxicity from concomitant patients. The joint evaluation of efficacy and safety was required due to the non-negligible toxicity risk of these anti-cancer agents [29, 30]. We assessed the operating characteristics of candidate designs and their robustness to departure from planned conditions and hypotheses.

Namely, we compared designs using standard binomial modelling of the study outcomes, either using Simon’s optimal design [26] or the Bayesian optimal design (BOP2 design) [23], combined with Bayesian posterior probability approaches for toxicity monitoring [24, 25], to designs incorporating time-to-event information on the outcomes, via a weighted likelihood, using the TOP design [22] (with and without informative prior from concomittant patients).

Overall, the TOP-design approaches showed greater power while controlling the type I error rate under the specified conditions prior to the trial, compared with BOP2 and Simon approaches. When considering toxicity monitoring, the posterior probability approach combined with TOP design had more power, but also was more subject to false positive in case of a toxic treatment. Conversely, using the TOP design for both efficacy and toxicity endpoints, an overestimated correlation between efficacy and toxicity at the planning stage can lead to a higher risk of false positive. In case of designs combining different approaches for efficacy and toxicity monitoring, the type I error rate increases with an overestimated correlation between efficacy and toxicity, but taking this correlation into account when planning the trial is not straightforward. Lastly, the addition of concomittant information for toxicity evaluation allowed a decreased proportion of false positive trials in case of a toxic treatment.

We focused on binding futility and toxicity rules, so the type I error rate was computed according to the decisions that were not ignored [31]. It is often preferred non-binding rules, especially for futility [32], as they provide more flexibility. Future work may address whether the nonbinding strategy increases or not the type I error rate and if corrections are needed to control it.

The TOP approaches represent an advantageous choice in clinical settings requiring long-term endpoints, which is frequent in oncology [6, 33, 34]. Compared to more complex approaches like the multiple-imputation one [35], TOP design would be simpler to implement and can save time in design calibration due to the closed-form of the posterior distribution. Subsequent prolonged observation windows [11, 13] can be challenging in designing early phase trials, particularly in the case of interim analyses. A major concern is then to determine when interim analyses will be performed. In the case of a design relying on strictly binary data for endpoints such as Simon’s optimal design, complete observations must be obtained to perform any analysis, potentially resulting in a waiting period with suspended inclusions over the course of the trial. The TOP design allows accounting for any available data at the time of an analysis, even incomplete observations, incorporating the fraction of available follow-up in the model via a weighted likelihood. Straightforwardly, the trial may allow continuous patient enrollment, and the trial duration can be reduced compared to a standard design. Moreover, in our setting, we found that the TOP approach was more powerful than the BOP, at the cost of a slight increase in the risk of false positives.

Concerning the observation window, safety monitoring approaches with the posterior probability of toxicity relied on binary data and therefore used complete toxicity observations only at the time of the enrollment of the next patient. In our settings, the observation window was shorter for toxicity (42 days) than for efficacy (6 months), while the anticipated average interpatient time was 6 days, for accrual. Should the toxicity observation window be longer and/or farther from the range of the interpatient time, given the default assumption of a uniform distribution of the toxicities over time, adapting the posterior probability approach with a time-to-event component, rather than excluding incomplete observations from interim analyses, should be considered to avoid false positive trials in case of toxic drugs. Indeed, the distribution of the time to toxicity may also affect the design’s operating characteristics. Moreover, an overly long observation window for both efficacy and toxicity may compromise the performances of the TOP design and lead to more statistical and organisational complexity. Future designs should avoid that and use reasonable observation windows to capture enough clinical information without leading to overly long trials.

Lastly, it has been shown that in the setting of frequentist approaches with efficacy stopping rule, more frequent looks can sometimes lead to an increased proportion of positive trials and thus to an overestimation of the efficacy, for example [36]. In our case, although we used Bayesian inference, we observed a similar outcome given more frequent looks and decisions; specifically, more frequent toxicity monitoring resulted in a greater probability of stopping for toxicity overall (data not shown).

## Conclusion

In our setting, we found that a design combining the TOP design for efficacy and posterior probability monitoring for toxicity results in greater power while controlling the risk of false positives. In cases where extra caution is needed due to a drug’s toxicity profile, a TOP design with joint efficacy and toxicity outcomes ensures a more conservative approach.

Furthermore, an advantage of the joint TOP approach for efficacy and toxicity is that it reduces the number of patients and the duration of the trial compared to posterior probability approaches, especially with prolonged toxicity observation windows.

Additionally, we found that concomitant data on toxicity, corresponding to a low-prevalence mutational subgroup in our setting, could be incorporated. In the case of Bayesian approaches, this is done through the toxicity informative prior, resulting in more stringent boundaries for safety assessment. Finally, one should be cautious about the correlation between efficacy and toxicity when planning a trial; however, the TOP design is more flexible and can accommodate different correlations if considered during planning.

## Supplementary Information

Additional file 1. Additional information and results.

## Data Availability

All codes to replicate datasets and analysis are available at https://github.com/GuillaumeMulier/MetimgastDesign.
