# Supplementary material for: Bayesian sequential monitoring strategies for trials of digestive cancer therapeutics
Source: BMC Med Res Methodol. 2024 Jul 19;24:154. doi: 10.1186/s12874-024-02278-3 (PMC11526600; doi:10.1186/s12874-024-02278-3)
Supplement: Supplementary file 1 — Additional file 1. Additional information and results. [file 12874_2024_2278_MOESM1_ESM.pdf]

Additional file 1 — Additional information and results

1 Calibration of designs

Calibration of PP_{tox} procedure

PP_{tox} procedure was calibrated for METIMGAST design so that in case of low toxicity (less than 20% toxicity rate), the type I error rate and the power of TOP-design was not much affected. In case of toxic treatement, the monitoring would lead to 49% of stopping for toxicity when the toxicity rate is 30%, and in 95% of stopping for toxicity when the toxicity rate is 40%.

Calibration of Simon's two-stage design

For comparative purposes, we also compared the different approaches with a Simon's two-stage design combined with the PP_{tox} toxicity monitoring (π_{tox}=0.25 and τ=0.95).

The settings used to optimize Simon's design were as follows: α=0.1, β=0.10, p_u=0.15, p_a=0.30. The optimal design enrolled 55 participants with an interim analysis at 23 patients. If fewer than 3 responses occurred at the first stage, the trial was stopped for futility, as well as if fewer than 11 patients experienced a response during the entire trial.

Because the other designs we evaluated had analyses at 30 and 81 patients for efficacy, we forced identical sample sizes for the analyses in Simon's design. Following the article by Simon in 1989, we determined optimal decision rules for this sample size, i.e., the design that minimizes the mean number of patients recruited in a trial when the treatment is not effective (with a probability of response equal to p_u). Thus, the trial was stopped for futility at the 1st stage if fewer than 5 responses occurred, and the treatment was considered unpromising if fewer than 16 responses occurred overall. This design (compared with the optimal design found without restrictions on the number of enrolled patients) had a type I error rate of 6.80% (vs 9.98%), a power of 91.0% (vs 90.1%), an expected mean sample size under p_u=0.15 of 44.8 patients (vs 37.7), and a probability of early termination under p_u of 71.1% (vs 54.0%).

Calibration for TOP design

As a sensitivity analysis, we conducted a few simulations to assess how the correlation between efficacy and toxicity might impact the calibration of the $TOP_{eff/tox}$ designs. Specifically, we recalibrated the parameters of the threshold for TOP designs on the same marginal working hypotheses but under different assumed correlations between efficacy and toxicity. Figure S1 illustrates the variation of the different design characteristics: type I error rate (denoted α) and power, according to the correlation between efficacy and toxicity, under $\{p_{0,\text{eff}} = 0.15; p_{0,\text{tox}} = 0.30\}$ and $\{p_{1,\text{eff}} = 0.30; p_{1,\text{tox}} = 0.20\}$. We found that the design's characteristics varied with the parameters of the thresholds calibrated under different correlations between efficacy and toxicity: while there was no clear pattern for the type I error rate, the power increased with the correlation under $\{p_{0,\text{eff}} = 0.15; p_{0,\text{tox}} = 0.30\}$ (power goes from around 85% at R_{min} to 95% at R_{max}). On the other side, the correlation under $\{p_{1,\text{eff}} = 0.30; p_{1,\text{tox}} = 0.20\}$ had less influence on the design's calibration and operating characteristics, with only slight changes in the expected power (power staying around 90% with the correlation of $R_{\text{pos},1}$ for H_0 and varying the correlation under H_1).

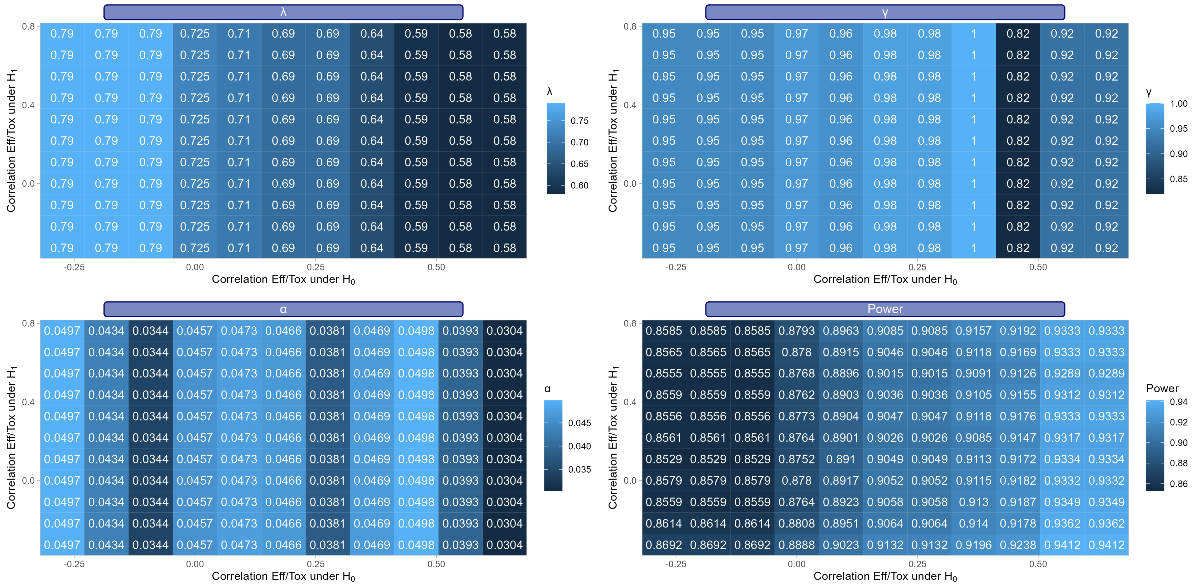

Figure S1: Heatmap of the thresholds and a priori error rate relative to the correlation between efficacy and toxicity under H_0 and H_1. The TOP-design was used with 2 interim analyses for efficacy and 10 for toxicity as defined above.

2 Correlation between efficacy and toxicity

Formula for the correlation between efficacy and toxicity :

$$R = \frac{P_{ET} - P_E P_T}{\sqrt{(P_E - P_E^2)(P_T - P_T^2)}}$$

with P_E the probability of efficacy, P_T the probability of toxicity and P_{ET} the probability of efficacy and toxicity.

The arbitrary values explored for the correlation between efficacy and toxicity are the following:

- R_{min} : When $Pr(\text{Eff} \cap \text{Tox}) = \max(0, Pr(\text{Eff}) + Pr(\text{Tox}) - 1)$;
- $R_{\text{neg}} = \frac{\text{R}_{\text{min}}}{2}$;
- $R_{\text{ind}} = 0$;
- $R_{\text{pos},1} = \frac{\text{R}_{\text{max}}}{3}$;
- $R_{\text{pos},2} = \frac{2\text{R}_{\text{max}}}{3}$;
- R_{max} : When $Pr(\text{Eff} \cap \text{Tox}) = \min(Pr(\text{Eff}), Pr(\text{Tox}))$.

3 Derivation of informative prior

Toxicity rates in BOP-design are analyzed as percentage and thus a conjugate beta prior is taken to derive the posterior distribution.

But observation windows can be prolonged and thus TOP-design made the hypothesis of a uniform distribution of toxicities during the observation window. Instead of planning the analysis after the observation window has been completed by a fixed number of patients, the analysis is

made after the number of patients have been recruited and the duration of observation is taken into account. Thus the posterior formula is adapted as follows :

$$Pr(\pi|D_n) \sim Beta(\alpha + \tilde{x}, \beta + ESS - \tilde{x})$$

with D_n the data available at the analysis, α and β are the parameters of the prior of π, $\tilde{x}$ the number of observed toxicities and ESS is the effective sample size.

The ESS represents a virtual number of patients observed by taking into account fully observed patients and pending patients.

$$ESS = \text{number of non-pending patients} + \frac{\sum \text{follow-up of pending patients}}{\text{number of pending patients} \times \text{maximal observation window}}$$

To derive the informative prior based on the cMET-positive cohort, we used the same formula and the prior parameters for toxicity rate α' and β' become $\alpha' = \frac{\alpha+\tilde{x}}{\kappa}$ and $\beta' = \frac{\beta+ESS-\tilde{x}}{\kappa}$. κ is a parameter to control the degree of borrowing from the additional cohort. We took $\kappa = 1$ as we took information from concomittant patients, but other values of κ can be explored based on sample size and origin of additionnal information; and can also be dynamically adapted.

4 Stopping rules

$\mathbf{TOP_{eff} + PP_{tox}}$

Decision rules for efficacy are the following:

Table 1: Stopping rules for futility of TOP_{eff} + PP_{tox} design.

Number of patients	Number of responses	Number of pending patients	Decision
30	≤ 3	≥ 12	Suspend inclusions
30	≤ 2	≤ 11	Stop for futility
30	3	≤ 11	Continue if ESS $<$ 22.72 else stop for futility
30	≥ 4	≤ 26	Continue
81	≤ 81	≥ 1	Suspend inclusions
81	≤ 16	0	Conclude to futility
81	≥ 17	0	Conclude to efficacy

Decision rules for toxicity are the following:

Table 2: Stopping rules for toxicity of $\text{TOP}_{\text{eff}} + \text{PP}_{\text{tox}}$ design.

Number of patients	Stop for toxicity if $>$ ... toxicities
5	2
10	4
15	6
20	8
30	11
40	14
50	17
60	20
70	23
80	26
90	29

Simon + PP_{tox}

Decision rules for efficacy are the following:

Table 3: Stopping rules for futility of Simon + PP_{tox} design.

Number of patients	Stop for futility if $\leq$ ... responses
30	5
81	16

Toxicity rules are the same as the $\text{TOP}_{\text{eff}} + \text{PP}_{\text{tox}}$ design and are thus show in table 2.

$\text{BOP}_{\text{eff}} + \text{PP}_{\text{tox}}$

Efficacy rules are the following:

Table 4: Stopping rules for futility of $\text{BOP}_{\text{eff}} + \text{PP}_{\text{tox}}$ design.

Number of patients	Stop for futility if $\leq$ ... responses
30	3
81	16

Toxicity rules are the same as the $\text{TOP}_{\text{eff}} + \text{PP}_{\text{tox}}$ design and are thus show in table 2.

$\mathbf{TOP_{eff/tox}}$ with close monitoring of toxicity

Futility rules are the following:

Table 5: Stopping rules for futility of $TOP_{eff/tox}$ design with close monitoring of toxicity.

Number of patients	Number of responses	Number of pending patients	Decision
30	≤ 3	≥ 12	Suspend inclusions
30	≤ 2	≤ 11	Stop for futility
30	3	≤ 11	Continue if ESS $<$ 25.9 else stop for futility
30	≥ 4	≤ 26	Continue
81	≤ 81	≥ 1	Suspend inclusions
81	≤ 14	0	Conclude to futility
81	≥ 15	0	Conclude to efficacy

Toxicity rules are the following:

Table 6: Stopping rules for toxicity of $\text{TOP}_{\text{eff/tox}}$ design with close monitoring of toxicity (1/2).

Patients	Toxicities	Pending patients	Toxic
5	≤ 3	≥ 1	Suspend inclusions
5	≤ 3	≤ 0	Continue
5	≥ 4	≤ 1	Stop for toxicity
10	≤ 5	≥ 2	Suspend inclusions
10	≤ 4	≤ 1	Continue
10	5	≤ 1	Stop for toxicity if ESS $\leq$ 9.52
10	≥ 6	≤ 4	Stop for toxicity
15	≤ 6	≥ 3	Suspend inclusions
15	≤ 5	≤ 2	Continue
15	6	≤ 2	Stop for toxicity if ESS $\leq$ 13.12
15	≥ 7	≤ 8	Stop for toxicity
20	≤ 8	≥ 5	Suspend inclusions
20	≤ 6	≤ 4	Continue
20	7	≤ 4	Stop for toxicity if ESS $\leq$ 16.85
20	8	≤ 4	Stop for toxicity if ESS $\leq$ 19.7
20	≥ 9	≤ 11	Stop for toxicity
30	≤ 10	≥ 12	Suspend inclusions
30	≤ 6	≤ 11	Continue
30	7	≤ 11	Stop for toxicity if ESS $\leq$ 18.57
30	8	≤ 11	Stop for toxicity if ESS $\leq$ 21.58
30	9	≤ 11	Stop for toxicity if ESS $\leq$ 24.6
30	10	≤ 11	Stop for toxicity if ESS $\leq$ 27.65
30	≥ 11	≤ 19	Stop for toxicity
40	≤ 13	≥ 20	Suspend inclusions
40	≤ 6	≤ 19	Continue
40	7	≤ 19	Stop for toxicity if ESS $\leq$ 20.13
40	8	≤ 19	Stop for toxicity if ESS $\leq$ 23.26
40	9	≤ 19	Stop for toxicity if ESS $\leq$ 26.4
40	10	≤ 19	Stop for toxicity if ESS $\leq$ 29.55
40	11	≤ 19	Stop for toxicity if ESS $\leq$ 32.71
40	12	≤ 19	Stop for toxicity if ESS $\leq$ 35.88
40	13	≤ 19	Stop for toxicity if ESS $\leq$ 39.06
40	≥ 14	≤ 26	Stop for toxicity
50	≤ 15	≥ 31	Suspend inclusions
50	≤ 6	≤ 30	Continue
50	7	≤ 30	Stop for toxicity if ESS $\leq$ 21.63
50	8	≤ 30	Stop for toxicity if ESS $\leq$ 24.87
50	9	≤ 30	Stop for toxicity if ESS $\leq$ 28.12
50	10	≤ 30	Stop for toxicity if ESS $\leq$ 31.38
50	11	≤ 30	Stop for toxicity if ESS $\leq$ 34.63
50	12	≤ 30	Stop for toxicity if ESS $\leq$ 37.89
50	13	≤ 30	Stop for toxicity if ESS $\leq$ 41.16
50	14	≤ 30	Stop for toxicity if ESS $\leq$ 44.42
50	15	≤ 30	Stop for toxicity if ESS $\leq$ 47.69
50	≥ 16	≤ 34	Stop for toxicity

Table 7: Stopping rules for toxicity of $TOP_{eff/tox}$ design with close monitoring of toxicity (2/2).

Patients	Toxicities	Pending patients	Toxic
60	$\leq$ 18	$\geq$ 45	Suspend inclusions
60	$\leq$ 4	$\leq$ 44	Continue
60	5	$\leq$ 44	Stop for toxicity if ESS $\leq$ 16.45
60	6	$\leq$ 44	Stop for toxicity if ESS $\leq$ 19.8
60	7	$\leq$ 44	Stop for toxicity if ESS $\leq$ 23.16
60	8	$\leq$ 44	Stop for toxicity if ESS $\leq$ 26.51
60	9	$\leq$ 44	Stop for toxicity if ESS $\leq$ 29.86
60	10	$\leq$ 44	Stop for toxicity if ESS $\leq$ 33.21
60	11	$\leq$ 44	Stop for toxicity if ESS $\leq$ 36.55
60	12	$\leq$ 44	Stop for toxicity if ESS $\leq$ 39.9
60	13	$\leq$ 44	Stop for toxicity if ESS $\leq$ 43.25
60	14	$\leq$ 44	Stop for toxicity if ESS $\leq$ 46.6
60	15	$\leq$ 44	Stop for toxicity if ESS $\leq$ 49.94
60	16	$\leq$ 44	Stop for toxicity if ESS $\leq$ 53.29
60	17	$\leq$ 44	Stop for toxicity if ESS $\leq$ 56.63
60	18	$\leq$ 44	Stop for toxicity if ESS $\leq$ 59.98
60	$\geq$ 19	$\leq$ 41	Stop for toxicity
70	$\leq$ 20	$\geq$ 61	Suspend inclusions
70	$\leq$ 2	$\leq$ 60	Continue
70	3	$\leq$ 60	Stop for toxicity if ESS $\leq$ 10.81
70	4	$\leq$ 60	Stop for toxicity if ESS $\leq$ 14.33
70	5	$\leq$ 60	Stop for toxicity if ESS $\leq$ 17.82
70	6	$\leq$ 60	Stop for toxicity if ESS $\leq$ 21.3
70	7	$\leq$ 60	Stop for toxicity if ESS $\leq$ 24.77
70	8	$\leq$ 60	Stop for toxicity if ESS $\leq$ 28.23
70	9	$\leq$ 60	Stop for toxicity if ESS $\leq$ 31.69
70	10	$\leq$ 60	Stop for toxicity if ESS $\leq$ 35.13
70	11	$\leq$ 60	Stop for toxicity if ESS $\leq$ 38.57
70	12	$\leq$ 60	Stop for toxicity if ESS $\leq$ 42.01
70	13	$\leq$ 60	Stop for toxicity if ESS $\leq$ 45.44
70	14	$\leq$ 60	Stop for toxicity if ESS $\leq$ 48.87
70	15	$\leq$ 60	Stop for toxicity if ESS $\leq$ 52.29
70	16	$\leq$ 60	Stop for toxicity if ESS $\leq$ 55.71
70	17	$\leq$ 60	Stop for toxicity if ESS $\leq$ 59.13
70	18	$\leq$ 60	Stop for toxicity if ESS $\leq$ 62.55
70	19	$\leq$ 60	Stop for toxicity if ESS $\leq$ 65.96
70	20	$\leq$ 60	Stop for toxicity if ESS $\leq$ 69.37
70	$\geq$ 21	$\leq$ 49	Stop for toxicity
81	$\leq$ 81	$\geq$ 1	Suspend inclusions
81	$\geq$ 23	0	Stop for toxicity
81	$\leq$ 22	0	Continue

5 Additionnal results

Operating characteristics

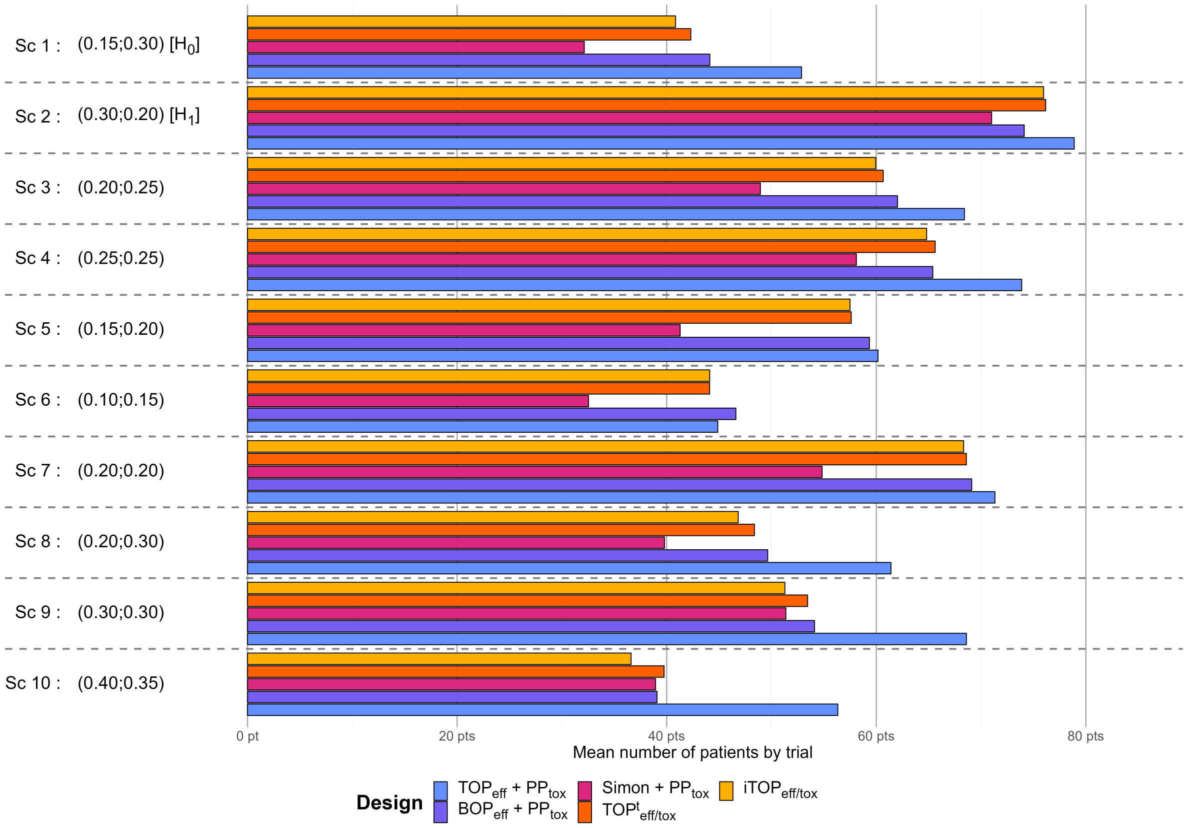

Figure S2: Average number of patients per trial in the 10 scenarios with a positive correlation between efficacy and toxicity. Numbers in parentheses represent p_{eff} and p_{tox} for each scenario.

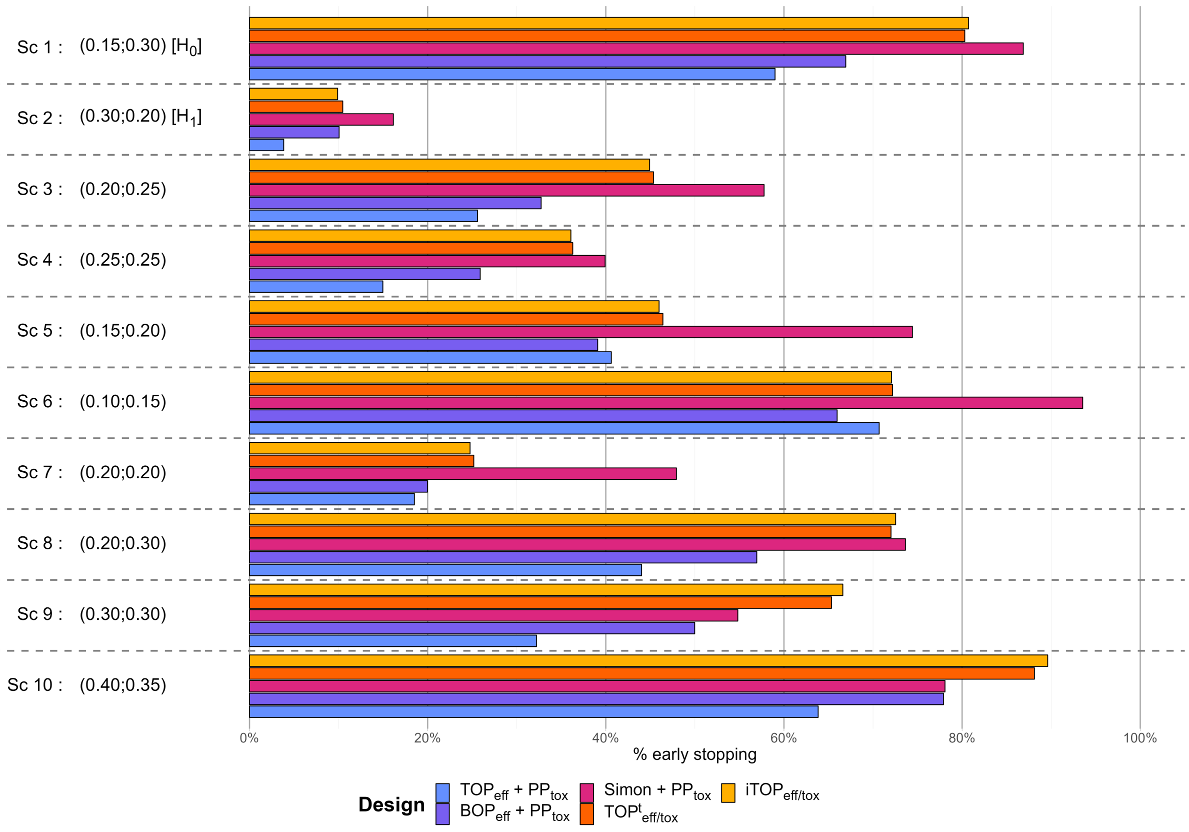

Figure S3: Proportion of early stopping in the 10 scenarios with a positive correlation between efficacy and toxicity. Numbers in parentheses represent p_{eff} and p_{tox} for each scenario.

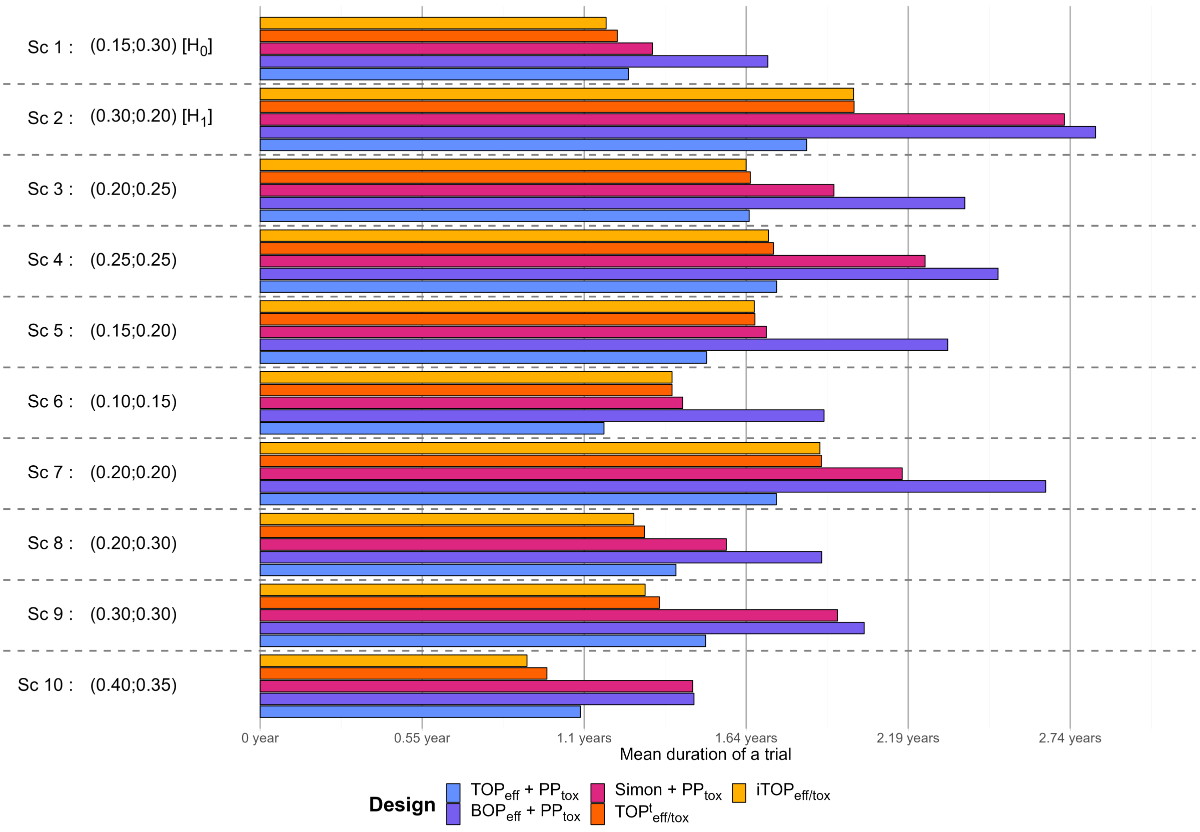

Figure S4: Mean duration of a trial in the 10 scenarios with a positive correlation between efficacy and toxicity. Numbers in parentheses represent p_{eff} and p_{tox} for each scenario.

Correlation between efficacy and toxicity

We report here the results under scenarios 1 to 10, with a varying correlation as described above, while the parameters were still optimized for a slight positive correlation (corresponding to $R_{\text{pos},1}$).

Scenario 1 shows the augmentation of the risk of false positive under the null hypothesis when the correlation between efficacy and toxicity decreases.In general, for all designs, when the correlation between efficacy and toxicity increases, the proportion of positive trials decreases in all scenarios except scenario 2. The effect of the correlation between efficacy and toxicity on the mean number of patients recruited is limited, but there is a decreased proportion of positive trials when the correlation between efficacy and toxicity increases. The same applies to the proportion of premature stopping and the mean duration of a trial. The overall conclusions are consistent with what was depicted for the positive correlation in the main article.

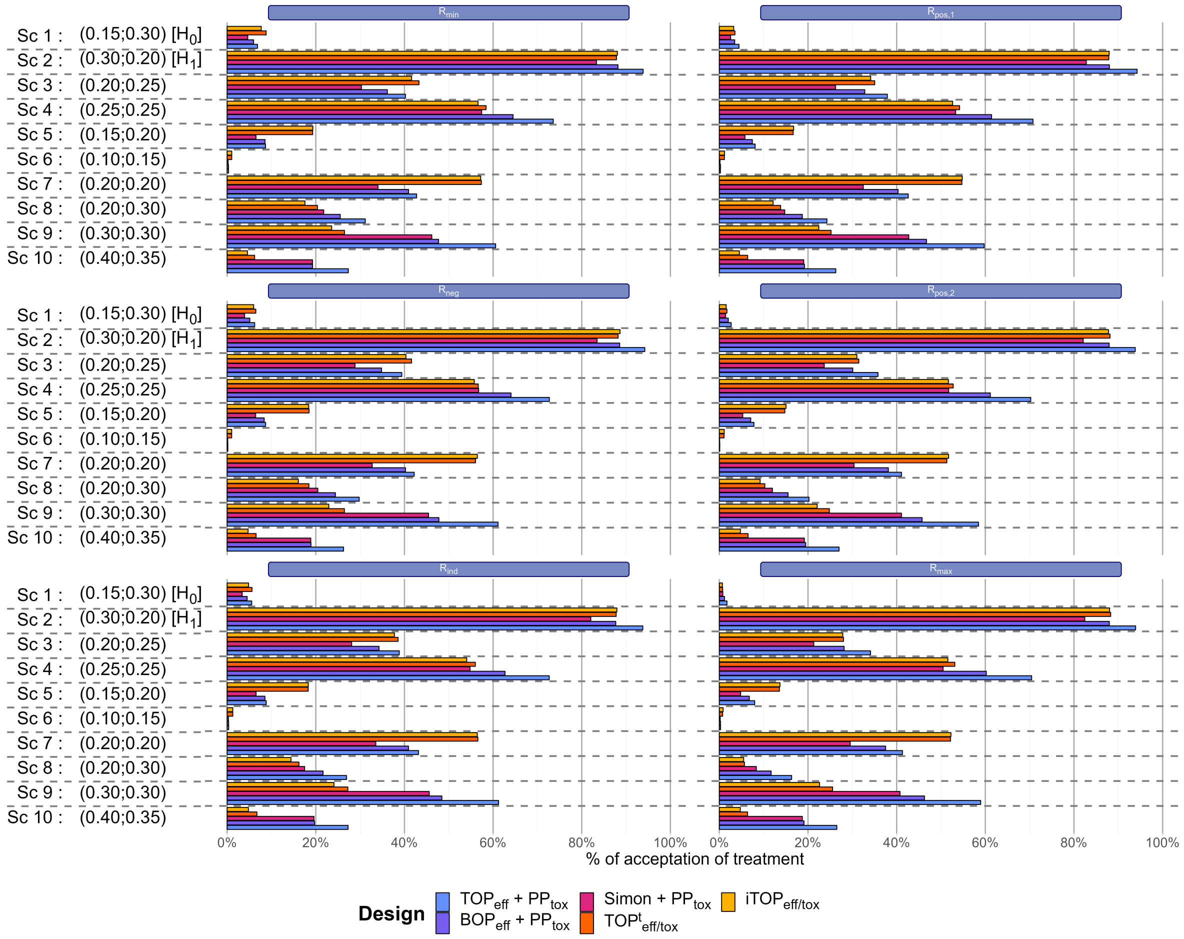

Figure S5: Percentage of conclusion of efficacy and acceptable toxicity in the 10 scenarios with 6 sets of correlation between efficacy and toxicity. Numbers in parentheses represent p_{eff} and p_{tox} for each scenario.

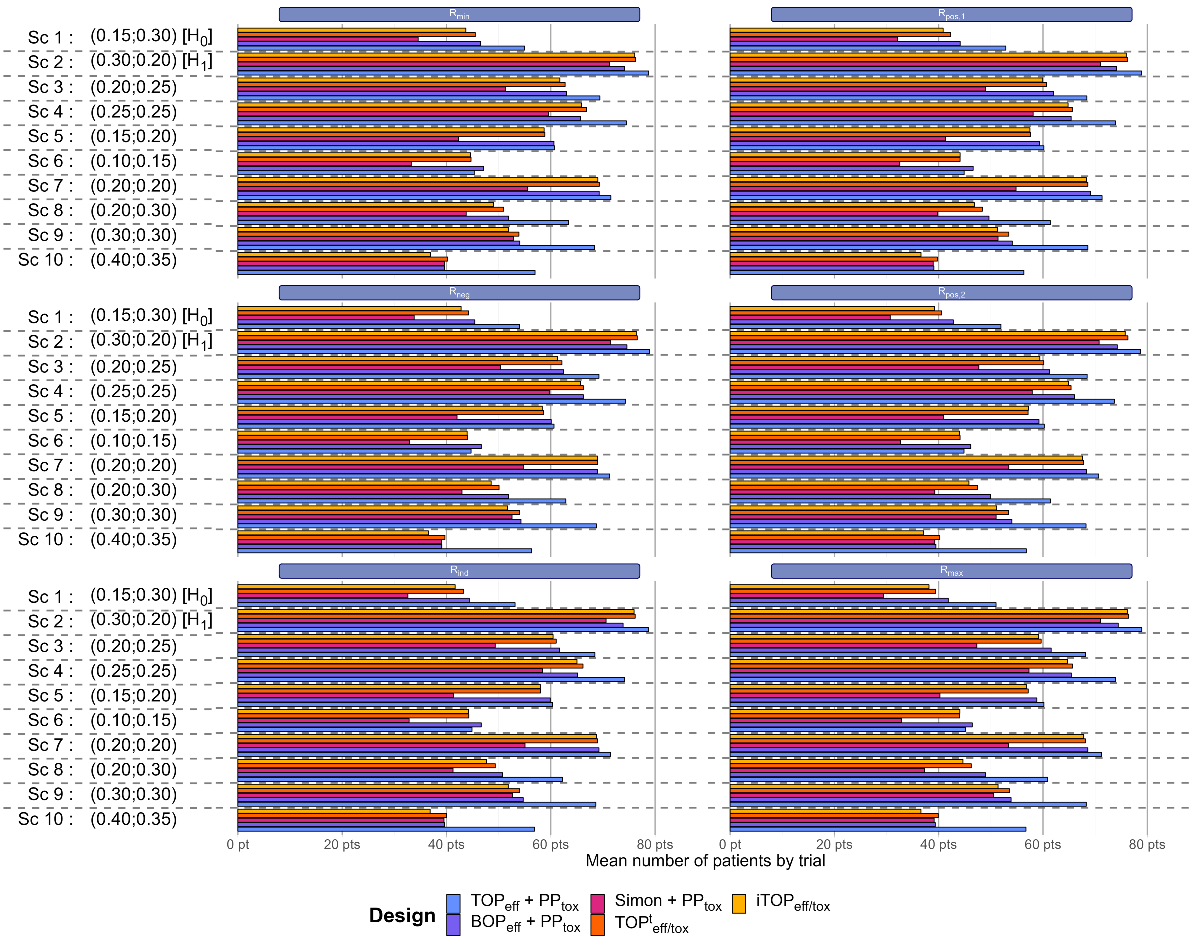

Figure S6: Mean number of patients per trial in the 10 scenarios with 6 sets of correlation between efficacy and toxicity. Numbers in parentheses represent p_{eff} and p_{tox} for each scenario.

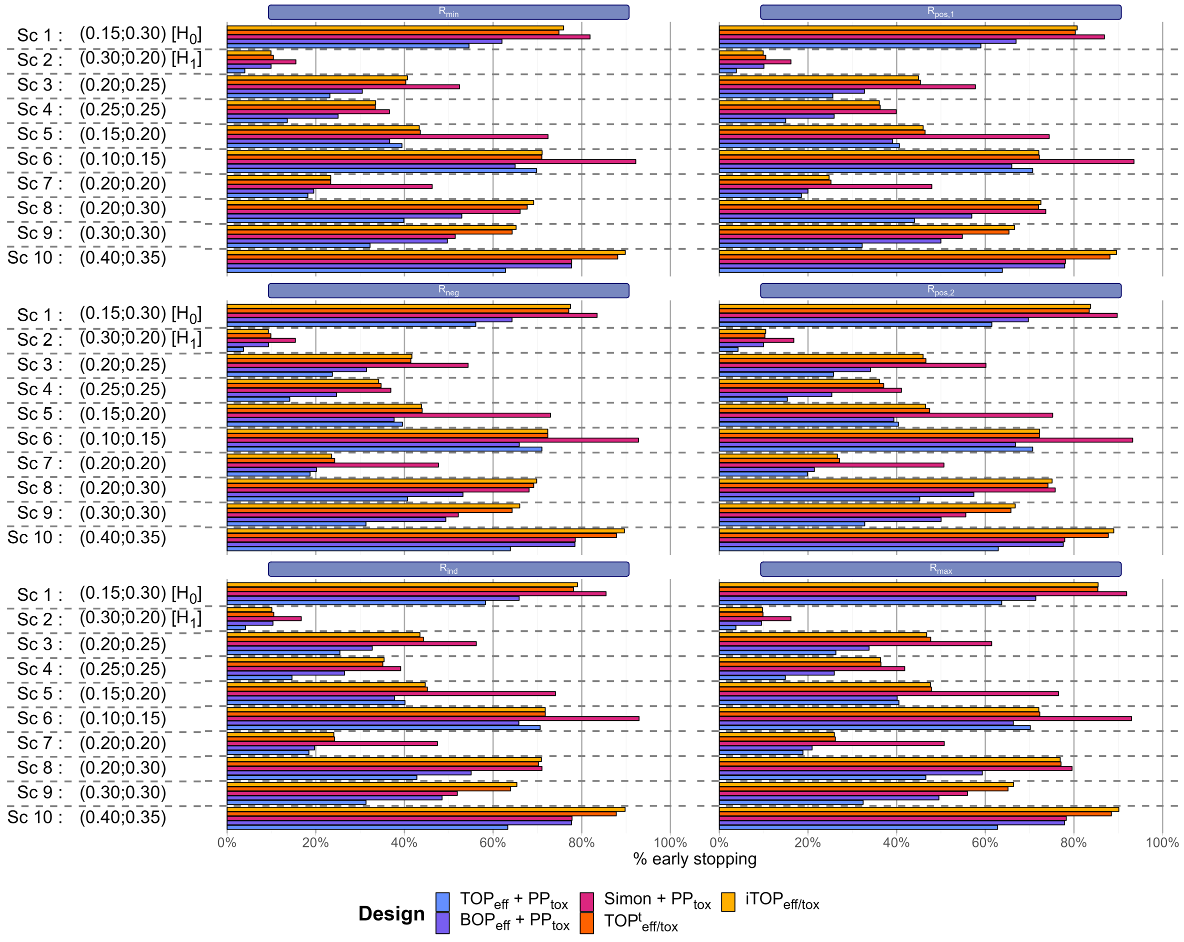

Figure S7: Proportion of early stopping in the 10 scenarios with 6 sets of correlation between efficacy and toxicity. Numbers in parentheses represent p_{eff} and p_{tox} for each scenario.

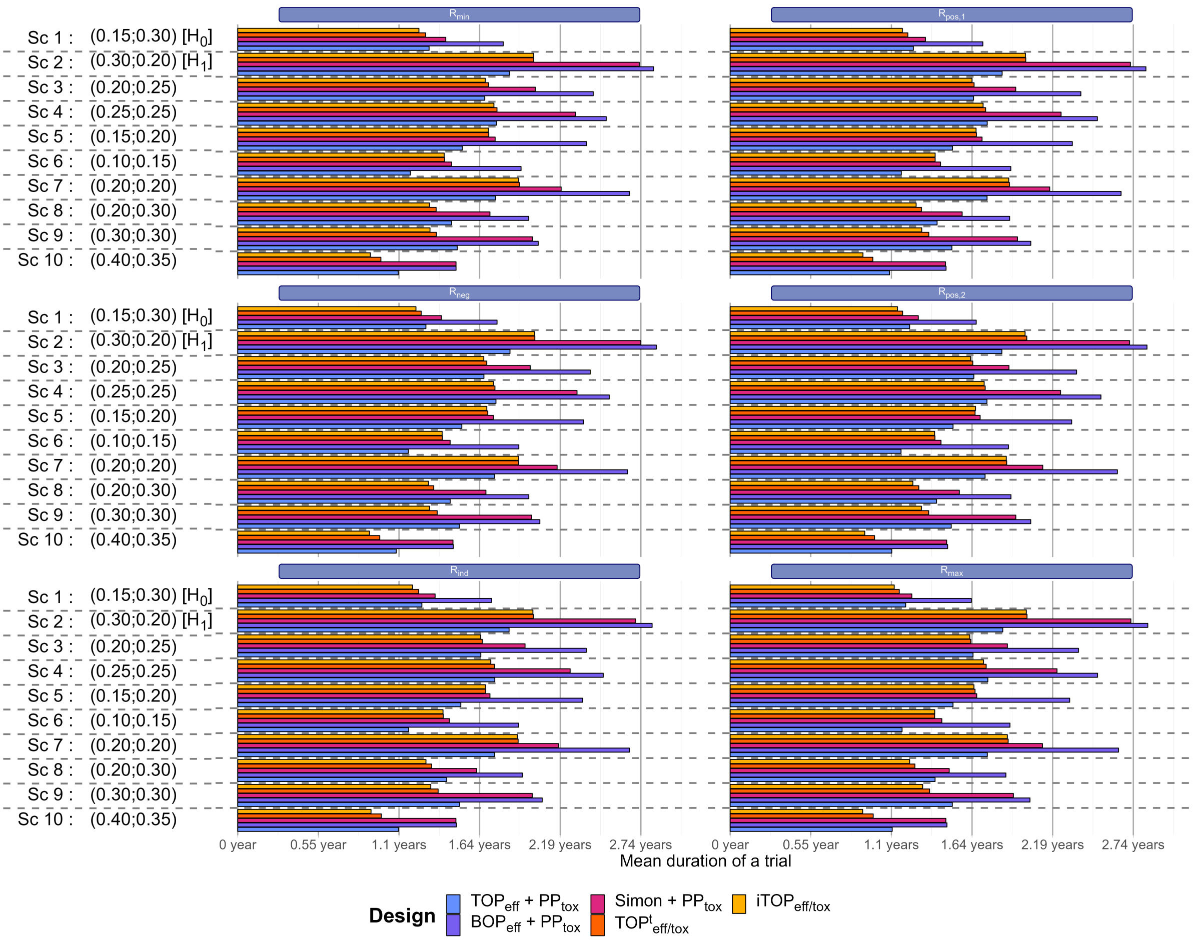

Figure S8: Mean duration of a trial in the 10 scenarios with 6 sets of correlation between efficacy and toxicity. Numbers in parentheses represent p_{eff} and p_{tox} for each scenario.
